# FGF21 signaling in glutamatergic neurons is required for weight loss associated with dietary protein dilution

**DOI:** 10.1038/s41598-020-76593-2

**Published:** 2020-11-11

**Authors:** Kyle H. Flippo, Sharon O. Jensen-Cody, Kristin E. Claflin, Matthew J. Potthoff

**Affiliations:** 1 Department of Neuroscience and Pharmacology, University of Iowa Carver College of Medicine, 169 Newton Road, 3322 PBDB, Iowa City, IA 52242 USA; 2 Fraternal Order of Eagles Diabetes Research Center, University of Iowa Carver College of Medicine, Iowa City, IA 52242 USA; 3 Iowa Neuroscience Institute, University of Iowa Carver College of Medicine, Iowa City, IA 52242 USA; 4 Department of Veterans Affairs Medical Center, Iowa City, IA 52242 USA

**Keywords:** Neuroscience, Physiology, Endocrinology, Molecular medicine

## Abstract

Alterations in macronutrient intake can have profound effects on energy intake and whole-body metabolism. For example, reducing protein intake increases energy expenditure, increases insulin sensitivity and decreases body weight in rodents. Fibroblast growth factor 21 (FGF21) signaling in the brain is necessary for the metabolic effects of dietary protein restriction and has more recently been proposed to promote protein preference. However, the neuron populations through which FGF21 elicits these effects are unknown. Here, we demonstrate that deletion of β-klotho in glutamatergic, but not GABAergic, neurons abrogated the effects of dietary protein restriction on reducing body weight, but not on improving insulin sensitivity in both diet-induced obese and lean mice. Specifically, FGF21 signaling in glutamatergic neurons is necessary for protection against body weight gain and induction of UCP1 in adipose tissues associated with dietary protein restriction. However, β-klotho expression in glutamatergic neurons was dispensable for the effects of dietary protein restriction to increase insulin sensitivity. In addition, we report that FGF21 administration does not alter protein preference, but instead promotes the foraging of other macronutrients primarily by suppressing simple sugar consumption. This work provides important new insights into the neural substrates and mechanisms behind the endocrine control of metabolism during dietary protein dilution.

## Introduction

All organisms must be able to sense environmental cues and respond to nutritional challenges or stress to maintain energy homeostasis. The ability to adaptively regulate macronutrient preference is especially important in mammals given their complex dietary needs requiring a proper balance of protein, carbohydrates, and fats for optimal physiological function^1^. However, the systems responsible for integrating nutrient status and macronutrient preference to prevent nutrient excess or deficiency are only recently becoming clear. For example, under conditions of amino acid restriction, mammals will increase intake of low protein diets to achieve physiologically necessary levels of dietary protein^2,3^, a phenomenon termed “the protein leverage effect”^4–10^. This increase in food intake arising from restriction of amino acids results in a coordinated increase in energy expenditure, likely to offset increased caloric intake and maintain body weight homeostasis. Numerous studies have revealed that the endocrine hormone fibroblast growth factor 21 (FGF21) production from the liver is required to mediate the protein leverage effect^11,12^. Specifically, FGF21 is required for the protection against weight gain, increased energy expenditure, and improved insulin sensitivity that is associated with dietary protein restriction^11–15^. Additionally, pharmacological administration of FGF21 has been proposed to enhance protein preference through the brain^16^, but questions still remain as to whether the effect of FGF21 on protein preference is a secondary consequence of FGF21′s effects on carbohydrate preference. In order for diets low in protein to achieve isocaloric density with control diets, the carbohydrate content is increased. Thus, while FGF21 can be induced by amino acid restriction independent of carbohydrate levels^13,17,18^, observed effects on diet preference may be due to differences in carbohydrate content as opposed to protein content. Physiologically, FGF21 is produced from the liver in response to various nutritional stresses (amino acid restriction and/or high carbohydrate intake)^19,20^. After entering circulation, FGF21 signals to cells which express the FGF21 co-receptor β-klotho (KLB) and FGFR1c^21–27^. KLB expression is restricted to a limited number of metabolic tissues and is absolutely required for FGF21 signaling^23,24,28,29^.

Recent work indicates that FGF21 signaling to the brain plays an important role in mediating the metabolic effects of FGF21 associated with dietary protein restriction^16^. However, it remains unclear what type of neurons FGF21 signals to in order to mediate these effects. To more explicitly define FGF21′s ability to modulate carbohydrate and protein preference we assessed FGF21′s effect on each alone and in combination. FGF21 does not appear to alter protein preference but robustly inhibits sugar preference, an effect we recently demonstrated requires KLB expression in glutamatergic neurons^30^. To determine the molecular identity of cells in the brain which FGF21 signaling is required for to mediate the effects of dietary protein restriction, we identified the molecular subtypes of neurons which express KLB. Based on those analyses we generated two novel mouse models in which FGF21′s obligate co-receptor, KLB, was deleted from neurons expressing either glutamate (Vglut2^+^, KLB Vglut2-KO) or GABA (Vgat^+^, KLB Vgat-KO). In this study, we find that KLB expression in glutamatergic neurons, but not GABAergic neurons, is required for protection against weight gain associated with dietary protein restriction. Interestingly, KLB expression in glutamatergic neurons is dispensable for the enhanced insulin sensitivity observed during dietary protein restriction. These findings identify the neuron population through which FGF21 mediates the metabolic effects of dietary protein restriction and supports previous work suggesting FGF21 mediated improvements in insulin sensitivity occur through peripheral targets^31^.

## Methods

All experiments presented in this study were conducted according to the animal research guidelines from NIH and were approved by the University of Iowa IACUC.

### Animals

The following mice were utilized in these studies (Jackson laboratory stock number in parenthesis): KLB^fl/fl22^, Vglut2-IRES-CRE (028863)^32^, and Vgat-IRES-CRE (028862)^32^. All mice were males and on a C57BL/6J genetic background. All mice used in experiments were individually housed under a 12 h light/dark cycle at 22–23 °C. Littermates were randomly assigned to experimental groups to achieve weight-matching between experimental groups. Animals were 9–12 weeks old at the start of each experiment. All animals used in this manuscript were not used for any other experiments. Health status was normal for all animals.

### Three-bottle choice experiments

For three-bottle choice experiments, drinking tubes were constructed and test fluids were presented following the Monell Mouse Taste Phenotyping Project specifications (https://www.monell.org/MMTPP/), and mice were offered the indicated test fluid versus water. Mice were individually housed and acclimated to regular handling with mock intraperitoneal (i.p.) injections for 4 days prior to the start of the experiment. Animals were injected with either vehicle or recombinant FGF21 (recombinant human FGF21, Novo Nordisk) at 1 mg/mL prior to accessing fluid solutions with ad-libitum access to normal chow (Teklad 2920×). Mice were administered i.p. injections of vehicle for 4 days, followed by FGF21 for 4 days and fluid intake was recorded daily. Injections of FGF21 or vehicle were performed at zeitgeber time (ZT) 7. Immediately following 4 days of FGF21 treatments, mice were subjected to a washout period in which they received no injections for 3 days and fluid intake was measured daily. The position of the fluid solutions were switched daily to prevent learning bias. Solutions were available 23 h/day and fluid intake was recorded and tubes were refilled during the remaining hour.

All fluid solutions were prepared with deionized water and served at room temperature. Animals were randomly assigned into groups of 8 and received their respective fluid solutions. All animals received 3 bottles/cage irrespective of the solutions presented. Animals in groups that received only sucrose (10% or 20%) or only casein (4% or 18%) were given two bottles with water and one bottle with sucrose or casein, respectively.

### Experimental diets

Mice were provided a control (20 kcal% protein) or low protein (5 kcal% protein) diet in the context of a normal chow or high fat diet. Customized diets were obtained from Research Diets: rodent diet with 10 kcal% fat (D12450B), rodent diet with 10 kcal% fat and 5 kcal% protein (D10062201), rodent diet with 60 kcal% fat (D12492), and rodent diet with 60 kcal% fat and 5 kcal% protein (D12020703). Diets were designed to be isocaloric by equally varying protein and carbohydrate while keeping fat constant. All diet compositions are provided in Supplementary Table 1. High-fat diets were provided to indicated mice to induce obesity. Individually housed mice were split into groups to ensure weight matching between treatment groups and placed on normal chow (NC), low protein (LP), high fat (HFD), or high fat low protein (HFLP) diets for 5 weeks. Food intake and body weight measurements were collected at the same time weekly over 5 weeks. Body composition and energy expenditure were measured via NMR and Promethion, respectively, and are detailed below. At the end of each study, mice were sacrificed via decapitation and trunk blood and tissues were collected. Trunk blood was centrifuged at 3000xg at 4 °C for serum collection. Tissue was snap frozen in liquid nitrogen immediately following collection for further analysis.

### In silico analysis of deposited single-cell RNA sequencing

Single cell RNA sequencing data of the whole hypothalamus was downloaded from GEO (accession number: GSE132730)^33^ and loaded into the R package Seurat (v3.1). *Klb* expressing cells in the “Neurons” sub-class of data were then isolated. Within the identified *Klb* expressing neurons the expression of each gene was scaled using the ScaleData function which scales the mean expression of each gene to the variance of its expression across all cells. Subclusters within *Klb*-expressing neurons were determined by identifying highly variable genes which was used as an input for dimensionality reduction via principle component analysis (PCA). The identified principle components were then used as an input for clustering analysis using the FindClusters function which identified 3 unique clusters within *Klb* neurons. Within subclustered *Klb* expressing neurons we evaluated expression of *Camk2a, Snap25, Vglut2* (*Slc17a6*), *Vgat* (*Slc32a1*), and *DAT* (*Slc6a3)*.

### Behavioral and metabolic phenotyping

Body composition was determined using a rodent sized nuclear magnetic resonance (NMR) machine (Bruker Minispec, LF50) as previously described^31^. Briefly, awake animals were lightly restrained in a polycarbonate tube during the 1–2 min recording and then immediately placed back into home cages. For mice lacking KLB expression in glutamatergic or GABAergic neurons (KLB Vglut2-KO or KLB Vgat-KO mice, respectively) receiving HFD and HFLP diets, body composition measurements were taken at week 0, 3, and 5. For KLB Vglut2-KO and KLB Vgat-KO mice receiving NC and LP diets, body composition measurements were taken at week 0 and week 5. Simultaneous determination of whole animal energy expenditure, respiratory exchange ratio, food intake, and physical activity was conducted using metabolic chambers (Promethion (Sable Systems International) for HFD and HFLP studies and CLAMS (Columbus Instruments) for NC and LP studies).

### Insulin tolerance tests

Insulin tolerance tests were performed at week 6 on indicated diets for lean mice and diet-induced obese mice. On the day of the experiment individually housed mice were fasted for 5 h with free access to water. Time 0 blood was collected at ZT 3 via tail bleed, followed by i.p. injections with human insulin (0.5 U insulin/kg body weight (BW) for lean mice and 0.75 U insulin/kg BW for DIO mice, Sigma, Cat#: I9278, Lot#: SLBN8658V) at ZT 3:15. Tail blood was collected into 300K2E microvettes (Sarstedt, #16.444.100) at the indicated times post-injection. Mice were maintained on their respective diets throughout experiments. Plasma glucose levels were subsequently measured with a colorimetric assay (Wako) using a Molecular Devices Spectra Max i3 to measure absorbance values according to the manufacturer’s instructions.

### Gene expression

RT-qPCR was used for assessment of mRNA levels in liver and adipose tissues (white and brown adipose tissue), and gene expression analyses were performed as described^31^. Briefly, RNA was isolated from the indicated tissues using Trizol (Invitrogen) reagent following the manufacture’s protocol. RNA purity and quantity were determined by spectrophotometry using a NanoDrop. 2 µg RNA from each sample was used to generate cDNA (High-Capacity cDNA Reverse Transcription Kit; Life Technologies), and qPCR was conducted using SYBR green (Invitrogen) and run on an Applied Biosystems 7900 HT RT-qPCR instrument. *U36B4*: 5′ -CGTCCTCGTTGGAGTGACA-3′, 5′ -CGGTGCGTCAGGGATTG-3′; *UCP1*: 5′-AAGCTGTGCGATGTCCATGT-3′, 5′-AAGCCACAAACCCTTTGAAAA-3′; and *Fgf21* FWD 5′-CCTCTAGGTTTCTTTGCCAACAG-3′, REV 5′-AAGCTGCAGGCCTCAGGAT-3'.

### Immunoassay determination of FGF21

Plasma levels of mouse FGF21 were determined using a commercially available ELISA (BioVendor). Blood was collected into 300K2E microvettes (Sarstedt, #16.444.100) and spun down at 3000 rpm for 30 min at 4 °C to collect plasma. Mouse FGF21 measurements were collected according to the procedure recommended by the manufacturer using a Molecular Devices Spectra Max i3 to measure absorbance values.

### Determination of plasma triglycerides and cholesterol

Plasma triglyceride and cholesterol levels were determined using commercially available kits (Thermo Scientific, Infinity Triglycerides (TR22421) and Infinity Cholesterol (TR13421), respectively). Blood was collected into 300K2E microvettes (Sarstedt, #16.444.100) and spun down at 3000 rpm for 30 min at 4 °C to collect plasma. Plasma triglyceride and cholesterol levels were measured according to the manufacturer’s instructions using a Molecular Devices Spectra Max i3 to measure absorbance values.

### Statistical analysis

Mice were assigned to groups based upon initial body mass for weight-matching. Where possible, analysis of data collection was blinded. Statistical analyses were performed using 2-way analysis of variance (ANOVA) with Holm–Sidak adjusted multiple comparisons unless otherwise stated. All analyses were carried out with GraphPad Prism v.7.01 (GraphPad Software, Inc.). Statistical details can be found within the figure legends. Differences between groups were considered significant when *P* < 0.05.

## Results

### FGF21 does not influence protein preference either alone or in combination with sucrose

**Figure 1:**
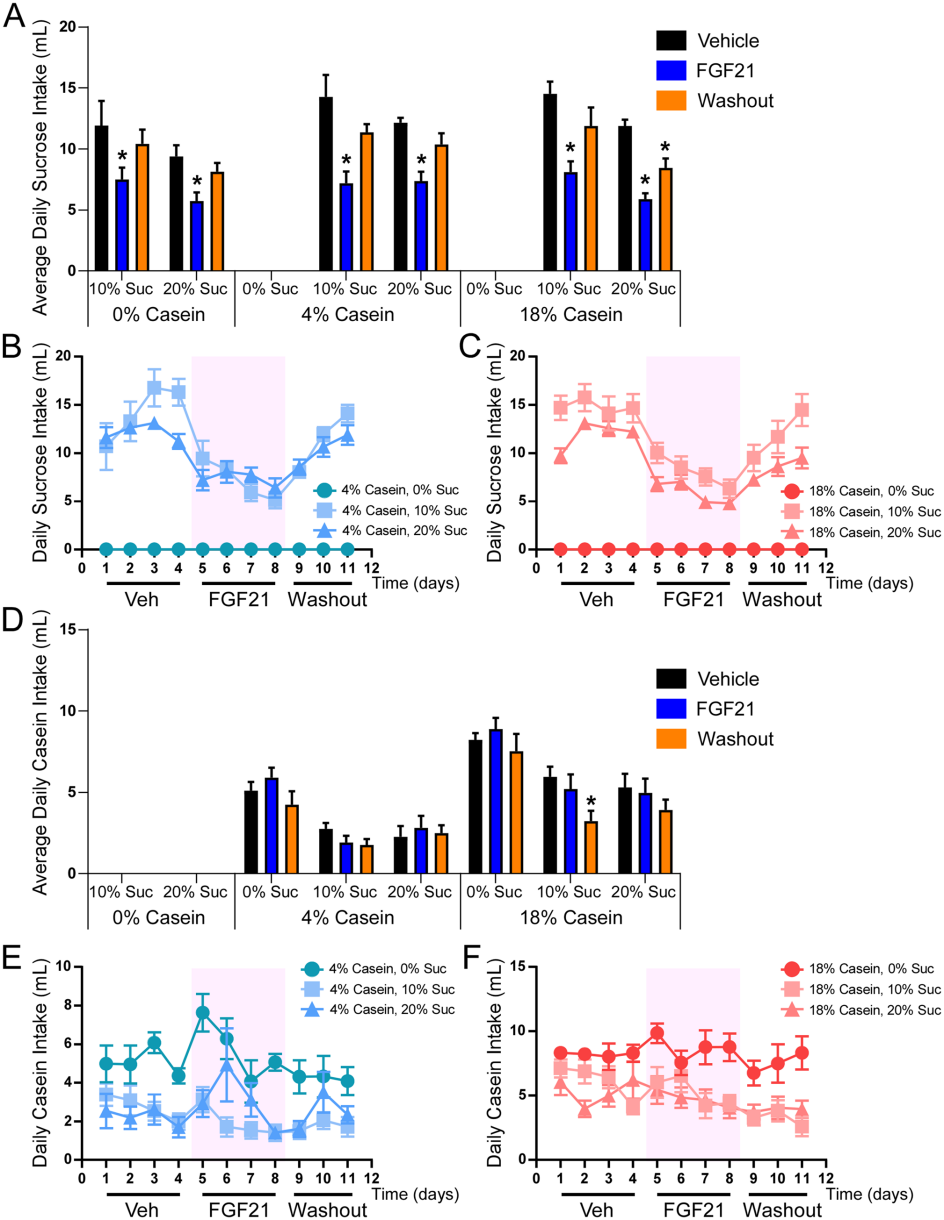
FGF21 does not influence protein preference either alone or in combination with sucrose. (**A**–**C**) Average daily sucrose intake during 3-bottle choice of water versus either 10% or 20% sucrose and/or 4% or 18% casein in 13-week old male wild-type (WT) mice receiving daily intraperitoneal (i.p.) injections of vehicle (4 days), followed by daily i.p. injections of FGF21 (1 mg/kg; 4 days), and then a washout period with no injections (3 days; n = 8/group). (**D**–**F**) Average daily casein intake during 3-bottle choice of water versus either 10% or 20% sucrose and/or 4% or 18% casein in 13-week old male WT mice receiving daily i.p. injections of vehicle (4 days), followed by daily i.p. injections of FGF21 (1 mg/kg; 4 days), and then a washout period with no injections (3 days; n = 8/group). Values are mean ± SEM. Statistical analyses were conducted using 2-way ANOVA with a Tukey multiple comparisons test, * = *P* < 0.05 compared with WT.

**Figure 2:**
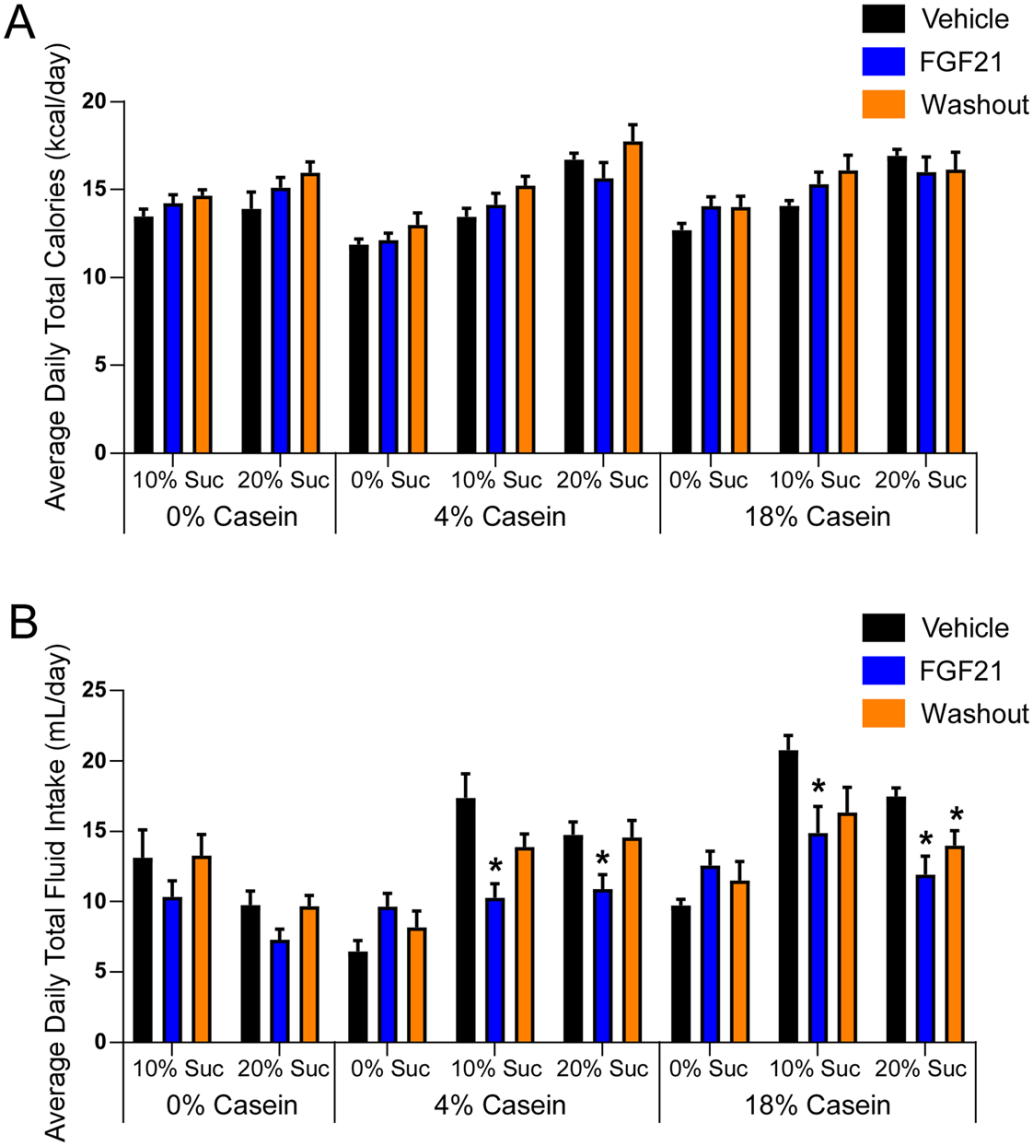
FGF21 decreases total fluid intake without altering total calories consumed. (**A**) Average daily total calories consumed (food and fluids) during 3-bottle choice of water versus either 10% or 20% sucrose and/or 4% or 18% casein in 13-week old male wild-type (WT) mice receiving daily intraperitoneal (i.p.) injections of vehicle (4 days), followed by daily i.p. injections of FGF21 (1 mg/kg; 4 days), and then a washout period with no injections (3 days; n = 8/group). (**B**) Average daily total fluid intake during 3-bottle choice of water versus either 10% or 20% sucrose and/or 4% or 18% casein in 13-week old male WT mice receiving daily i.p. injections of vehicle (4 days), followed by daily i.p. injections of FGF21 (1 mg/kg; 4 days), and then a washout period with no injections (3 days; n = 8/group). Values are mean ± SEM. Statistical analyses were conducted using 2-way ANOVA with a Tukey multiple comparisons test, * = *P* < 0.05 compared with WT.

FGF21 regulates macronutrient preference through direct signaling to the brain. While multiple studies have demonstrated that FGF21 suppresses sugar intake and sweet taste preference^34–36^, the effect of FGF21 on protein preference has been less clear. To thoroughly address this question, we performed a combination of three-bottle preference tests in which mice were given a choice between water, varying concentrations of sucrose solutions (10% and 20% solutions), and/or normal and low casein solutions (18% and 4%, respectively). Fluid intake of each of the three bottles and food intake were measured in mice receiving daily vehicle injections for four days followed by daily FGF21 injections for four days and then a washout period of 3 days. Similar to our previous studies, FGF21 suppressed sucrose consumption at all sucrose concentrations independent of whether sucrose was presented with water alone or under conditions of water and casein (Fig. 1A–C). Sucrose consumption returned to normal in mice in all groups by the third day of the washout period. Notably, we observed no significant effect of FGF21 on casein consumption over the treatment period (Fig. 1D). While protein consumption increased transiently with FGF21 administration in two of the 4% casein groups, this effect was not maintained throughout the treatment period (Fig. 1E), as was observed for sucrose (Fig. 1B,C), and was not observed in the 18% casein group (Fig. 1F). In addition, no change between the end of the treatment period and washout period was observed (Fig. 1E,F). Importantly, consistent with our previous work, acute FGF21 administration (≤ 3 days) had no effect on total caloric intake (Fig. 2A). In addition, FGF21 administration had no effect on total protein calories consumed when casein was available (Supplementary Fig. 1A,C,E). Under various conditions, FGF21 reduced total fluid intake (Fig. 2B) which was attributable to the marked decrease in sucrose consumption in response to FGF21 (Fig. 1A–C; Supplementary Fig. 1B,D,F). Together, these data indicate that FGF21′s primary and sustained effect on macronutrient preference is to regulate sugar, not protein, intake.

### FGF21 signaling in glutamatergic neurons is required to protect against weight gain during dietary protein restriction in DIO mice

**Figure 3:**
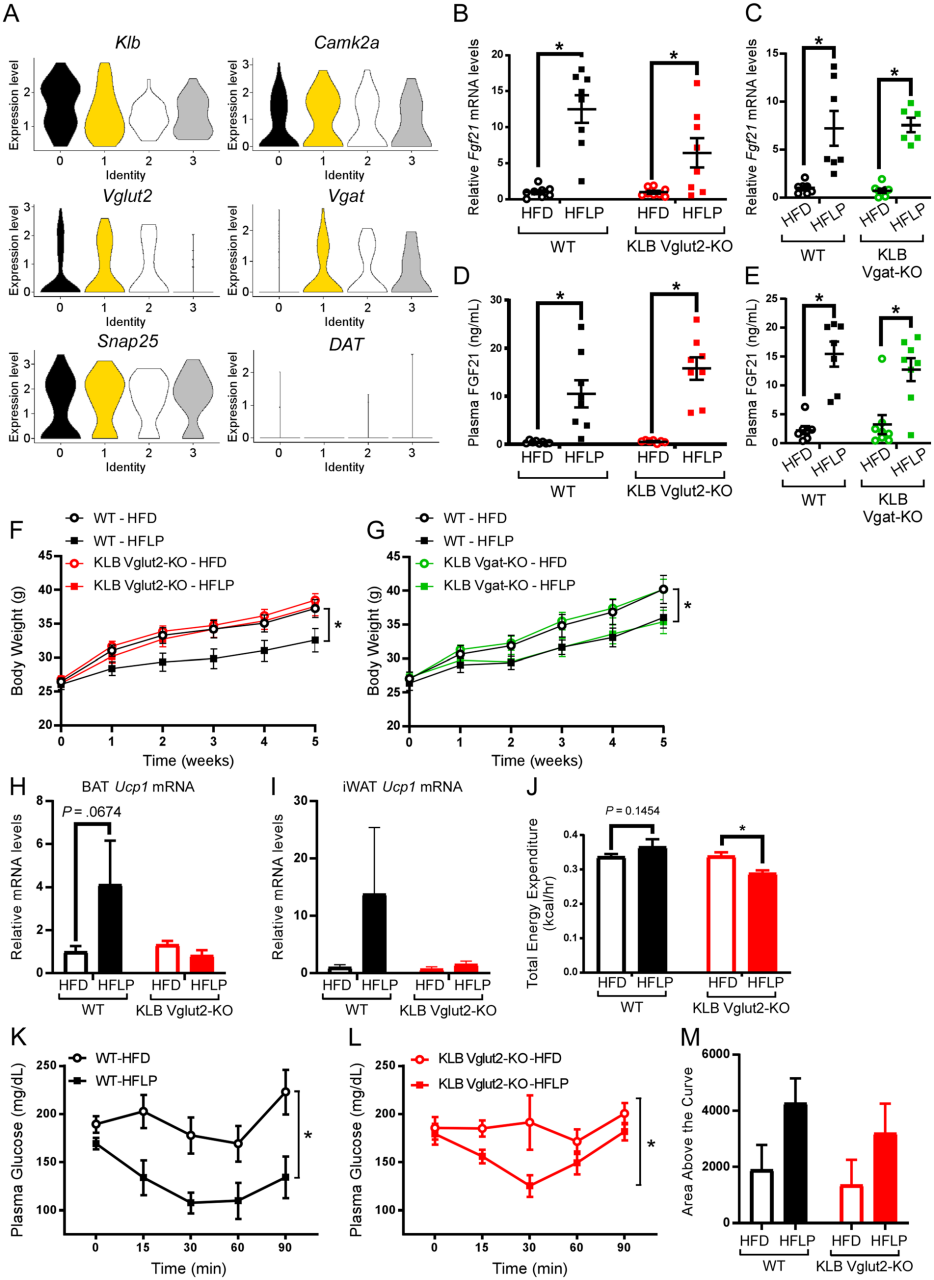
FGF21 signaling in glutamatergic, but not GABAergic, neurons is required to protect against weight gain during dietary protein restriction in DIO mice. (**A**) Violin plots of *in-silico* single cell RNA sequencing analysis of relative mRNA expression of indicated molecular identifiers in *Klb* expressing neurons isolated from the hypothalamus (n = 183 neurons). (**B**,**C**) Relative *Fgf21* mRNA expression in the liver of wild-type (WT) mice and mice lacking β-klotho (KLB) in (**B**) Vglut2-expressing cells (KLB Vglut2-KO mice) or (**C**) Vgat-expressing cells (KLB Vgat-KO mice) following 5 weeks on high fat diet (HFD) or high fat, low protein diet (HFLP) (n = 6–8 mice/group). (**D**,**E**) Circulating FGF21 protein levels in plasma from (**D**) WT and KLB Vglut2-KO mice or (**E**) WT and KLB Vgat-KO mice following 5 weeks on HFD or HFLP (n = 7–8 mice/group). (**F**) Body weight curves of WT and KLB Vglut2-KO mice on HFD or HFLP over 5 weeks (n = 11–15 mice/group). (**G**) Body weight curves of WT and KLB Vgat-KO mice on HFD or HFLP over 5 weeks (n = 8–10 mice/group). (**H**,**I**) Relative *Ucp1* mRNA levels in brown adipose tissue (BAT) (**H**) and inguinal white adipose tissue (iWAT) (**I**) of wild-type (WT) mice and mice lacking β-klotho (KLB) in Vglut2-expressing cells (KLB Vglut2-KO mice) on high fat diet (HFD) or high fat, low protein diet (HFLP) (n = 7–8 mice/group). (**J**) Total energy expenditure (EE) in WT and KLB Vglut2-KO mice on HFD or HFLP measured by indirect calorimetry (n = 8 mice/group). (**K**,**L**) Plasma glucose levels during an insulin tolerance test (ITT) in (K) WT and (L) KLB Vglut2-KO mice, respectively, on HFD or HFLP (n = 6 mice/group). (M) Quantification of the average area above the curve for the ITTs plotted in (**K**) and (**L**). Values are mean ± SEM. 2-way ANOVA with Holm-Sidak’s multiple comparisons test performed for all panels with mice on HFD used as the control condition within genotypes for statistical comparisons, * = *P* < 0.05.

FGF21 signaling to KLB-expressing cells in the central nervous system (CNS) is required for the protection against weight gain attributed to dietary protein restriction^16^. Our recent work indicates FGF21 signals directly to KLB-expressing glutamatergic neurons to suppress carbohydrate intake^30^. However, it has yet to be investigated what types of neurons FGF21 signals to in order to elicit the beneficial metabolic effects of dietary protein restriction. To begin to identify what type(s) of cells FGF21 may signal to in order to mediate the metabolic effects of dietary protein restriction, we performed in silico analysis of available single cell RNA sequencing data from neurons in the hypothalamus^33^, a brain region well known for its role in regulating energy expenditure and macronutrient preference. Initially, we profiled which types of cells expressed KLB (KLB^+^) and observed strong expression in neurons (data not shown). To follow up, we analyzed KLB^+^ neuronal populations by sub-clustering KLB^+^ neurons (confirmed by evaluating expression of *Camk2a* and *Snap25*).We found that KLB^+^ neurons largely express the vesicular glutamate transporter, *Vglut2* (*Slc17a6*), or the vesicular GABA transporter, *Vgat* (*Slc32a1*), each representative markers of glutamatergic and GABAergic neurons, respectively (Fig. 3A). Interestingly, we found that KLB^+^ neurons exhibit sparse expression of the dopamine transporter, DAT (Fig. 3A), a marker of dopamine producing neurons.

To determine whether FGF21 signaling in glutamatergic or GABAergic neurons contributes functionally to the metabolic effects of dietary protein restriction, we deleted KLB in either glutamatergic (KLB Vglut2-KO) or GABAergic (KLB Vgat-KO) expressing neurons by crossing KLB^fl/fl^ mice to Vglut2-IRES-CRE or Vgat-IRES-CRE mice, respectively. All mice were then placed on either a traditional high fat diet (HFD; 60% fat) or a high fat, low protein diet (HFLP; 60% fat, 5% protein) for 5 weeks and body weight and food intake were measured. Consistent with previous studies, we observed a marked increase in hepatic and plasma FGF21 levels in wild-type (WT) mice consuming a HFLP diet compared to HFD-fed mice (Fig. 3B–E). In addition, as expected, hepatic and plasma levels of FGF21 were similarly induced in KLB Vglut2-KO (Fig. 3B,D) and KLB Vgat-KO mice (Fig. 3C,E) on a HFLP diet compared to WT littermate controls on a HFLP diet. Importantly, when comparing the effects of HFD and HFLP in WT mice we observed the expected protection against weight gain and increase in food intake^16^, suggestive of increased metabolic capacity, associated with HFLP feeding (Fig. 3F,G and Supplementary Fig. 2A,B). Notably, however, the capacity of low protein to protect against body weight gain was completely lost in KLB Vglut2-KO mice (Fig. 3F) but was fully retained in KLB Vgat-KO mice (Fig. 3G). This protection against body weight gain by HFLP feeding occurred despite a significant increase in food intake in WT mice (Supplementary Fig. 2A,B). Together, these results demonstrate that KLB expression in Vglut2^+^ neurons is required for FGF21 to protect against body weight gain and to increase food intake during dietary protein restriction in diet-induced obese mice.

FGF21′s metabolic effects in response to dietary protein restriction are dependent upon induction of uncoupling protein 1 (UCP1)^15^. To evaluate whether deletion of KLB in Vglut2^+^ neurons may block the induction of UCP1 associated with increased circulating FGF21 during dietary protein restriction, we measured *Ucp1* mRNA levels in brown adipose tissue (BAT) and inguinal white adipose tissue (iWAT). As expected, we observed an increase in *Ucp1* mRNA expression in adipose tissues of WT mice receiving a HFLP diet relative to WT mice on HFD, but this effect was blocked in KLB Vglut2-KO mice (Fig. 3H,I). To determine whether there are functional changes in energy expenditure associated with increases in UCP1 expression, we measured energy expenditure in WT and KLB Vglut2-KO mice on either HFD or HFLP diet by indirect calorimetry. WT mice receiving HFLP diet trended to increase energy expenditure relative to WT HFD-fed mice (Fig. 3J). In contrast, we observed a significant reduction in energy expenditure in KLB Vglut2-KO mice on HFLP diet relative to KLB Vglut2-KO mice on HFD (Fig. 3J). We observed no effect of diet on water consumption (Supplementary Fig. 2C), plasma triglycerides (Supplementary Fig. 2D), or cholesterol (Supplementary Fig. 2E). Taken together, these results indicate that loss of FGF21 signaling to Vglut2^+^ neurons blocks the metabolic effects of dietary protein restriction on increasing energy expenditure and preventing body weight gain.

### The insulin sensitizing effects of low protein diets are maintained in DIO mice lacking FGF21 signaling to Vglut2^+^neurons

In addition to protecting against weight gain, dietary protein restriction also robustly increases insulin sensitivity which is dependent upon FGF21 expression in the liver^12^. Recent work also suggests that deletion of KLB in the brain may block the insulin sensitizing effects of dietary protein restriction^16^. Thus, we sought to explore whether the effects of dietary protein restriction on insulin sensitivity were maintained in KLB Vglut2-KO mice under DIO conditions. We therefore performed insulin tolerance tests (ITT) in WT and KLB Vglut2-KO mice fed either HFD or HFLP diet. Importantly, WT mice fed a HFLP diet exhibited a marked increase in insulin sensitivity compared to WT HFD-fed mice (Fig. 3K). Importantly, despite being resistant to low protein mediated reductions in body weight, the insulin sensitizing effects of dietary protein restriction were preserved in KLB Vglut2-KO mice fed HFLP diet compared to KLB Vglut2-KO mice fed HFD (Fig. 3L,M). In addition, deletion of KLB in GABAergic neurons did not block the effects of dietary protein restriction on insulin sensitivity (Supplementary Fig. 2F). These data suggest that the insulin sensitizing effects of dietary protein restriction do not require FGF21 signaling in glutamatergic or GABAergic neurons and exist independent of changes in body weight.

### FGF21 signaling in glutamatergic neurons is required to protect against weight gain during dietary protein restriction in lean mice

**Figure 4:**
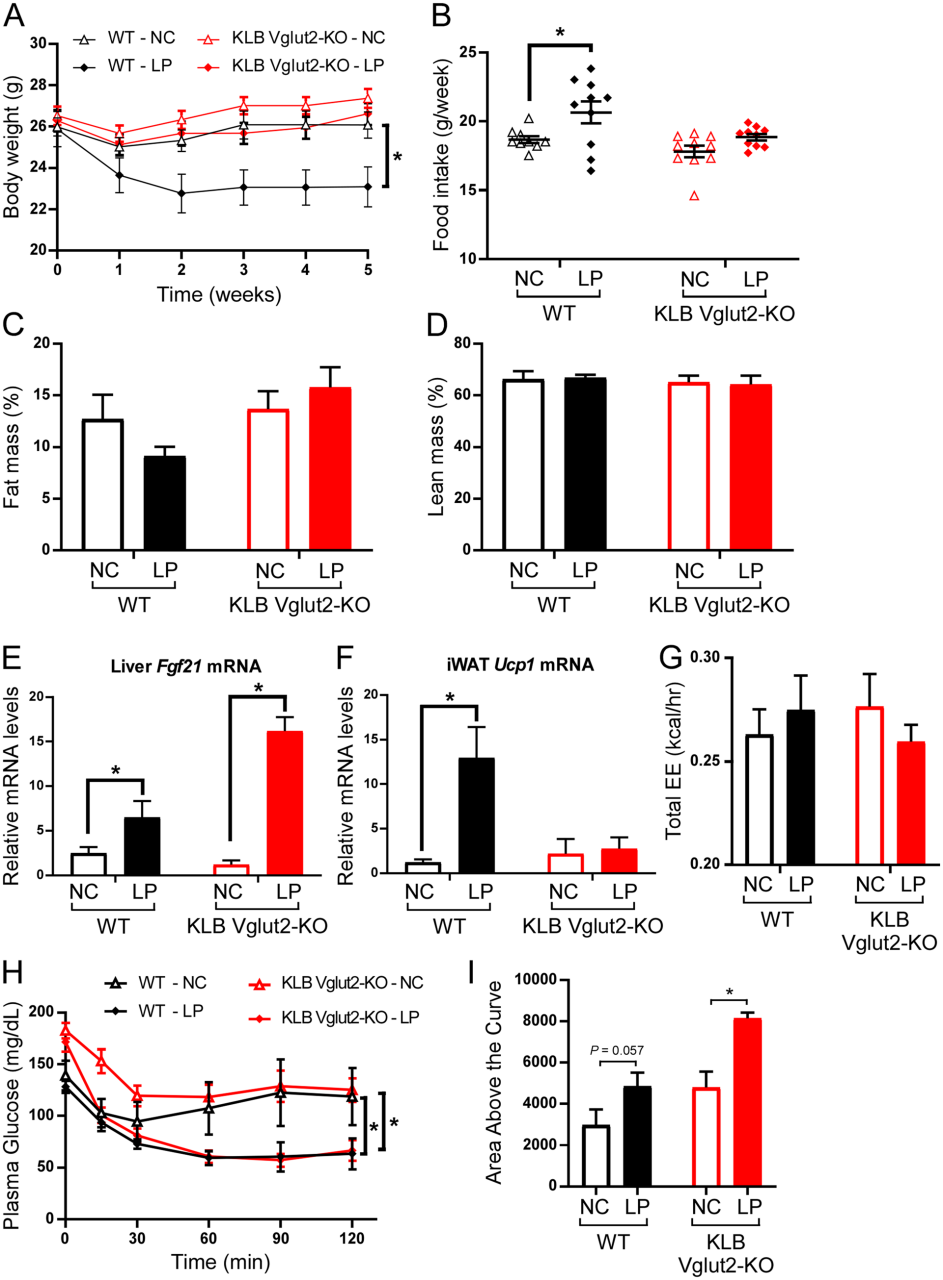
Loss of KLB in glutamatergic neurons in lean mice blocks the effects on body weight, but not insulin sensitivity associated with dietary protein restriction. (**A**) Body weight curves of wild-type (WT) mice and mice lacking β-klotho (KLB) in Vglut2-expressing cells (KLB Vglut2-KO mice) on normal chow (NC) or low protein chow (LP) over 5 weeks (n = 9–10 mice/group). (**B**) Average weekly food intake of WT and KLB Vglut2-KO mice on NC or LP. (**C**,**D**) Percentage of fat mass (**C**) and lean mass (**D**) in WT and KLB Vglut2-KO mice after 5 weeks on NC or LP (n = 5 mice for each group). (**E**) Relative *Fgf21* mRNA levels in the liver of WT and KLB Vglut2-KO mice following 5 weeks on NC or LP (n = 8–9 mice/group). (**F**) Relative *Ucp1* mRNA expression in inguinal white adipose tissue (iWAT) of WT and KLB Vglut2-KO mice on NC or LP (n = 9 mice/group). (**G**) Total energy expenditure (EE) in WT and KLB Vglut2-KO mice on NC or LP determined by indirect calorimetry (n = 8 mice/group). (**H**) Plasma glucose levels during an insulin tolerance test (ITT) in WT and KLB Vglut2-KO mice on NC or LP (n = 6 mice/group). (**I**) Quantification of the average area above the curve for the ITT curves plotted in (**H**). Values are mean ± SEM. 2-way ANOVA with Holm-Sidak’s multiple comparisons test performed for all panels with mice on NC used as the control condition within genotypes for statistical comparisons, * = *P* < 0.05.

To determine whether FGF21 signaling to glutamatergic neurons is also important for the metabolic effects of dietary protein restriction under conditions of normal caloric intake (i.e., in lean mice), we provided KLB Vglut2-KO and control littermates normal chow (NC) or low protein (LP, 5% protein) chow diets. Consistent with the data from our HFLP studies, WT mice receiving a LP diet exhibited decreased body weight and increased food intake relative to WT mice on NC. This effect was completely blocked in KLB Vglut2-KO mice (Fig. 4A,B). Similar to WT mice fed HFLP, lean WT mice on LP trended to have reduced fat mass compared to NC-fed control mice, an effect not seen in KLB Vglut2-KO mice (Fig. 4C). Alternatively, there was no difference in lean mass in either WT or KLB Vglut2-KO mice fed NC or LP diets (Fig. 4D). As expected, LP increased liver *Fgf21* expression in both WT and KLB Vglut2-KO mice (Fig. 4E). Similar to KLB Vglut2-KO mice fed HFLP, loss of KLB in Vglut2^+^ neurons blocked the induction of iWAT *Ucp1* expression by LP in lean KLB Vglut2-KO mice (Fig. 4F). However, we observed no effect on energy expenditure in these mice (Fig. 4G). Consistent with the results in DIO mice (Fig. 3K–M), the insulin sensitizing effects of dietary protein restriction were preserved in lean KLB Vglut2-KO mice, as they exhibit a similar decrease in plasma glucose levels as WT mice during an ITT, relative to WT and KLB Vglut2-KO mice on normal chow (Fig. 4H,I). Together, these data support our results in DIO mice and demonstrate the function of FGF21 signaling in glutamatergic neurons to mediate the metabolic effects, but not insulin sensitizing effects, of dietary protein restriction.

## Discussion

In this work, we provide important new insights into the neural substrates mediating the beneficial effects of dietary protein dilution and establish the importance of these neurons in regulating energy expenditure, insulin sensitivity and protein intake. We demonstrate that FGF21 signaling specifically to glutamatergic, but not GABAergic, neurons is required for the increases in energy expenditure and weight loss associated with low protein diet consumption. A requirement for FGF21 signaling to glutamatergic neurons in the ventromedial hypothalamus (VMH) was also recently demonstrated for its ability to regulate sugar intake^30^. However, in that same study FGF21 signaling to neurons in the VMH was not required for FGF21-mediated protection against weight gain^30^. Thus, together these data suggest that FGF21 regulates sugar consumption and body weight through spatially distinct populations of KLB^+^ glutamatergic neurons. Interestingly, we also reveal that while FGF21 signaling to glutamatergic neurons is required to mediate protection against weight gain associated with dietary protein restriction, FGF21 signaling to glutamatergic neurons is dispensable for dietary protein restriction-mediated improvements in insulin sensitivity in lean and DIO mice. In contrast to our data, previous work found that the insulin sensitizing effects of HFLP are blocked in mice lacking KLB expression in the brain^16^. Our data instead indicate that the insulin sensitizing effects of dietary protein restriction is maintained in mice lacking functional FGF21 signaling to glutamatergic neurons. Given the effects of low protein diet on body weight and insulin sensitivity, however, other tests, including hyperinsulinemic-euglycemic clamps, may provide better insights into the effect of diet on insulin sensitivity in the different genetic models. While we cannot exclude the possibility that other non-glutamatergic neuron populations may contribute to FGF21′s insulin sensitizing effects, we and others have previously demonstrated that the insulin sensitizing effects of FGF21 are mediated by adipose tissues^31,36,37^. Thus, our findings identify dissociable aspects of the metabolic effects of dietary protein restriction and suggest FGF21 signaling is required in multiple tissues to prevent metabolic dysfunction during macronutrient imbalance.

In addition to energy expenditure and body weight regulation, FGF21 induction by low protein diets has been proposed to regulate protein intake^16,38^. Our three-bottle choice experiments with different protein and carbohydrate concentrations revealed that FGF21 primarily regulates simple sugar intake, not protein preference. A previous study by Hill et al. observed an increase in casein intake in response to a single ICV injection of FGF21^16^. However, while we did observe a transient increase in protein intake in a couple of the 4% casein groups administered FGF21, the transient increase in protein intake in our studies was not consistent or sustained and, in some cases, was also observed in the washout period. In contrast, sucrose intake was significantly suppressed during FGF21 administration in all groups receiving sucrose and the response was reversed during the washout period. In addition, a previous study by Larson et al. compared FGF21-mediated preferences in diets with different macronutrient composition^38^. However, this approach is not adequate to accurately assess taste preferences^39,40^. Instead, our studies more comprehensively assess taste preferences mediated by FGF21 through fluid choice. While taste preference is only one aspect of macronutrient preference, the experimental paradigm used in our studies allows us to investigate macronutrient preference without manipulating diet composition, and therefore avoids the complications associated with it. That is, altering diet composition, for example to attain a low protein diet, not only reduces one macronutrient (i.e., protein) but also increases another macronutrient (i.e., carbohydrate), thereby complicating the interpretation of simple dietary manipulations^20^. In contrast, the fluid choice paradigm allows preference for specific macronutrients to be evaluated. A potential limitation of this approach, however, is that the macronutrients are presented in an arguably unnatural fashion (i.e., as liquids). Consistent with our data, though, administration of an FGF21 analog to obese subjects decreased preference for sweet tasting food and carbohydrate intake^41^. Moreover, the physiological relevance of FGF21 in regulating carbohydrate intake is demonstrated by single nucleotide polymorphisms (SNPs) at the FGF21 locus associating with increased intake of sweets^42^ and carbohydrates^43,44^. Finally, since FGF21 administration during this experiment did not alter total calorie intake, the increase in food intake observed during chronic dietary protein dilution is likely mediated by indirect mechanisms signaling increased fuel demands resulting from increased energy expenditure. Together, these data reveal that FGF21 functions directly to suppress carbohydrate intake, increase energy expenditure, and increase insulin sensitivity but does not significantly regulate protein or total calorie intake. Future work will focus on identifying the region of the brain in which KLB-expressing glutamatergic neurons reside to mediate FGF21′s metabolic effects during dietary protein restriction.

## Supplementary information

Supplementary Information
