# Supplementary material for: FGF21 signaling in glutamatergic neurons is required for weight loss associated with dietary protein dilution
Source: Sci Rep. 2020 Nov 11;10:19521. doi: 10.1038/s41598-020-76593-2 (PMC7658965; doi:10.1038/s41598-020-76593-2)
Supplement: Supplementary file 1 — Supplementary Information [file 41598_2020_76593_MOESM1_ESM.pdf]

Supplemental Materials

FGF21 signaling in glutamatergic neurons is required for weight loss associated with dietary protein dilution

Kyle H. Flippo[1,2,3,*], Sharon O. Jensen-Cody[1,2,3,*], Kristin E. Claflin[1,2,3], Matthew J. Potthoff[1,2,3,4,#]

[1]Department of Neuroscience and Pharmacology, University of Iowa Carver College of Medicine, Iowa City, IA 52242, USA.
[2]Fraternal Order of Eagles Diabetes Research Center, University of Iowa Carver College of Medicine, Iowa City, IA 52242, USA.
[3]Iowa Neuroscience Institute, University of Iowa Carver College of Medicine, Iowa City, IA 52242, USA.
[4]Department of Veterans Affairs Medical Center, Iowa City, IA 52242, USA.

SUPPLEMENTARY FIGURE LEGENDS

Supplementary Figure 1. Caloric consumption of protein and carbohydrate in food vs. fluid in mice from Figure 1 and 2. (A) Average daily total protein caloric intake (food + fluid) during 3-bottle choice of water versus either 10% or 20% sucrose and/or 4% or 18% casein in 13-week old male wild type (WT) mice receiving daily intraperitoneal (i.p.) injections of vehicle (4 days), followed by daily i.p. injections of FGF21 (1 mg/kg; 4 days), and then a washout period with no injections (3 days; n = 8/group). (B) Average daily total carbohydrate caloric intake (food + fluid) during 3-bottle choice of water versus either 10% or 20% sucrose and/or 4% or 18% casein in 13-week old male WT mice receiving daily i.p. injections of vehicle (4 days), followed by daily i.p. injections of FGF21 (1 mg/kg; 4 days), and then a washout period with no injections (3 days; n = 8/group). (C) Average daily protein caloric intake from food alone during 3-bottle choice of water versus either 10% or 20% sucrose and/or 4% or 18% casein in 13-week old male WT mice receiving daily i.p. injections of vehicle (4 days), followed by daily i.p. injections of FGF21 (1 mg/kg; 4 days), and then a washout period with no injections (3 days; n = 8/group). (D) Average daily carbohydrate caloric intake from food alone during 3-bottle choice of water versus either 10% or 20% sucrose and/or 4% or 18% casein in 13-week old male WT mice receiving daily i.p. injections of vehicle (4 days), followed by daily i.p. injections of FGF21 (1 mg/kg; 4 days), and then a washout period with no injections (3 days; n = 8/group). (E) Average daily protein caloric intake from casein alone during 3-bottle choice of water versus either 10% or 20% sucrose and/or 4% or 18% casein in 13-week old male WT mice receiving daily i.p. injections of vehicle (4 days), followed by daily i.p. injections of FGF21 (1 mg/kg; 4 days), and then a washout period with no injections (3 days; n = 8/group). (F) Average daily carbohydrate caloric intake from sucrose alone during 3-bottle choice of water versus either 10% or 20% sucrose and/or 4% or 18% casein in 13-

week old male WT mice receiving daily i.p. injections of vehicle (4 days), followed by daily i.p. injections of FGF21 (1 mg/kg; 4 days), and then a washout period with no injections (3 days; n = 8/group). Values are mean +/- SEM. 2-way ANOVA with Holm-Sidak's multiple comparisons test performed for all panels with vehicle treatment used as the control condition for statistical comparisons, * = $P < 0.05$.

Supplementary Figure 2. Increased food intake associated with dietary protein dilution is blocked in mice lacking functional FGF21 signaling to glutamatergic neurons without altering water consumption or plasma lipid levels. (A) Weekly food intake on high fat diet (HFD) or high fat, low protein (HFLP) in wild type (WT) and mice lacking β-klotho in Vglut2-expressing cells (KLB Vglut2-KO mice) over 5 weeks (n = 11-15 mice/group). (B) Weekly food intake on HFD or HFLP in WT mice and mice lacking KLB in Vgat-expressing cells (KLB Vgat-KO mice) over 5 weeks (n = 8-10 mice/group). (C) Average daily water consumption measured in metabolic chambers in WT and KLB Vglut2-KO mice on HFD or HFLP for 5 weeks (n = 8 mice/group). (D) Serum triglyceride levels measured in WT and KLB Vglut2-KO mice on HFD or HFLP after 5 weeks on the respective diets (n = 8 mice/group). (E) Serum cholesterol levels measured in WT and KLB Vglut2-KO mice on HFD or HFLP after 5 weeks on the respective diets (n = 8 mice/group). (F) Plasma glucose levels during an insulin tolerance test (ITT) in WT mice and KLB Vgat-KO mice on HFD or HFLP for 5 weeks (n = 6-8 mice/group). Values are mean +/- SEM. 2-way ANOVA with Holm-Sidak's multiple comparisons test performed for all panels with mice on HFD used as the control condition within genotypes for statistical comparisons, * = $P < 0.05$.

Supplementary Table 1. Composition of diets related to Methods. Composition of all diets for animal studies are as indicated.

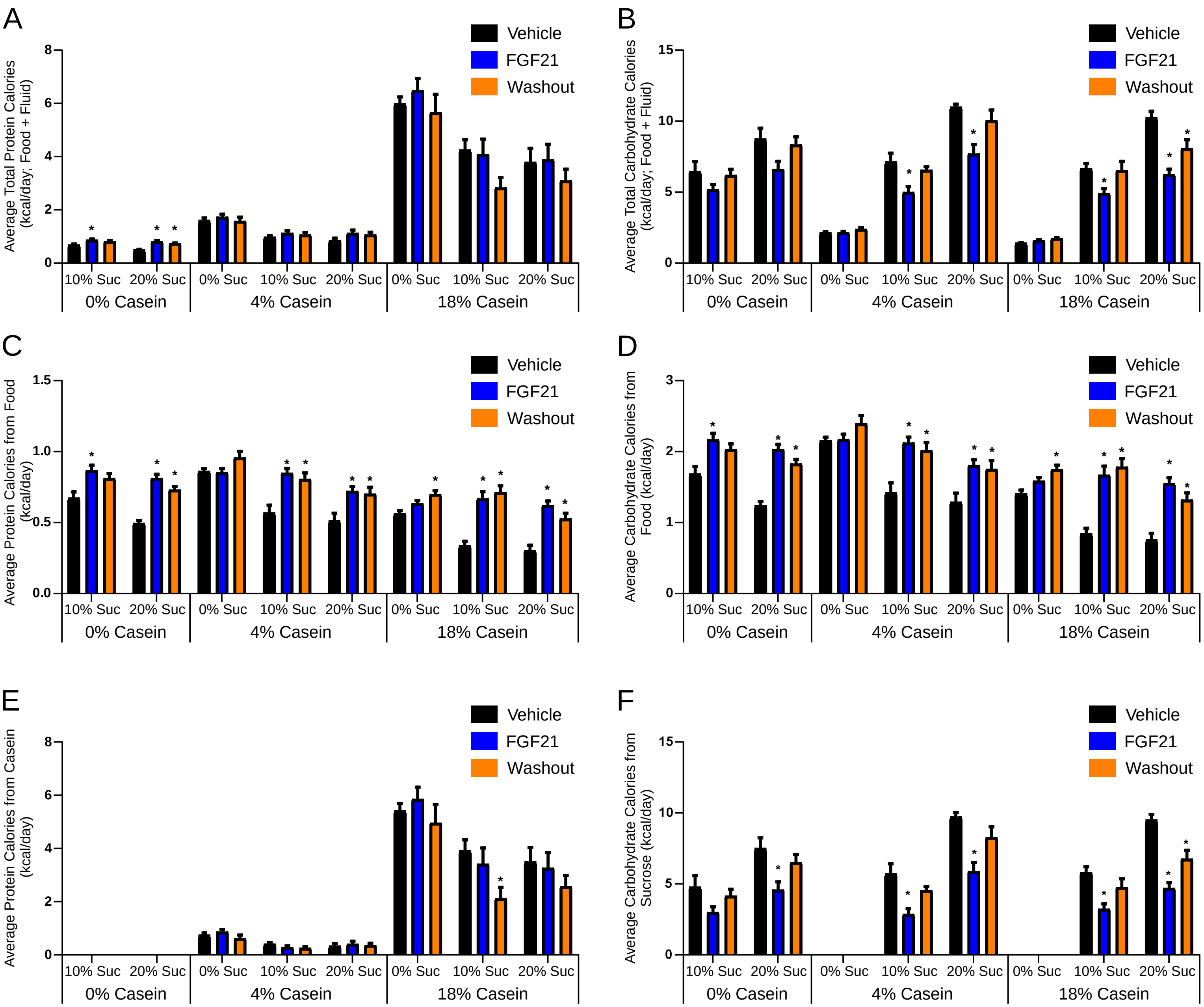

Supplementary Figure 1

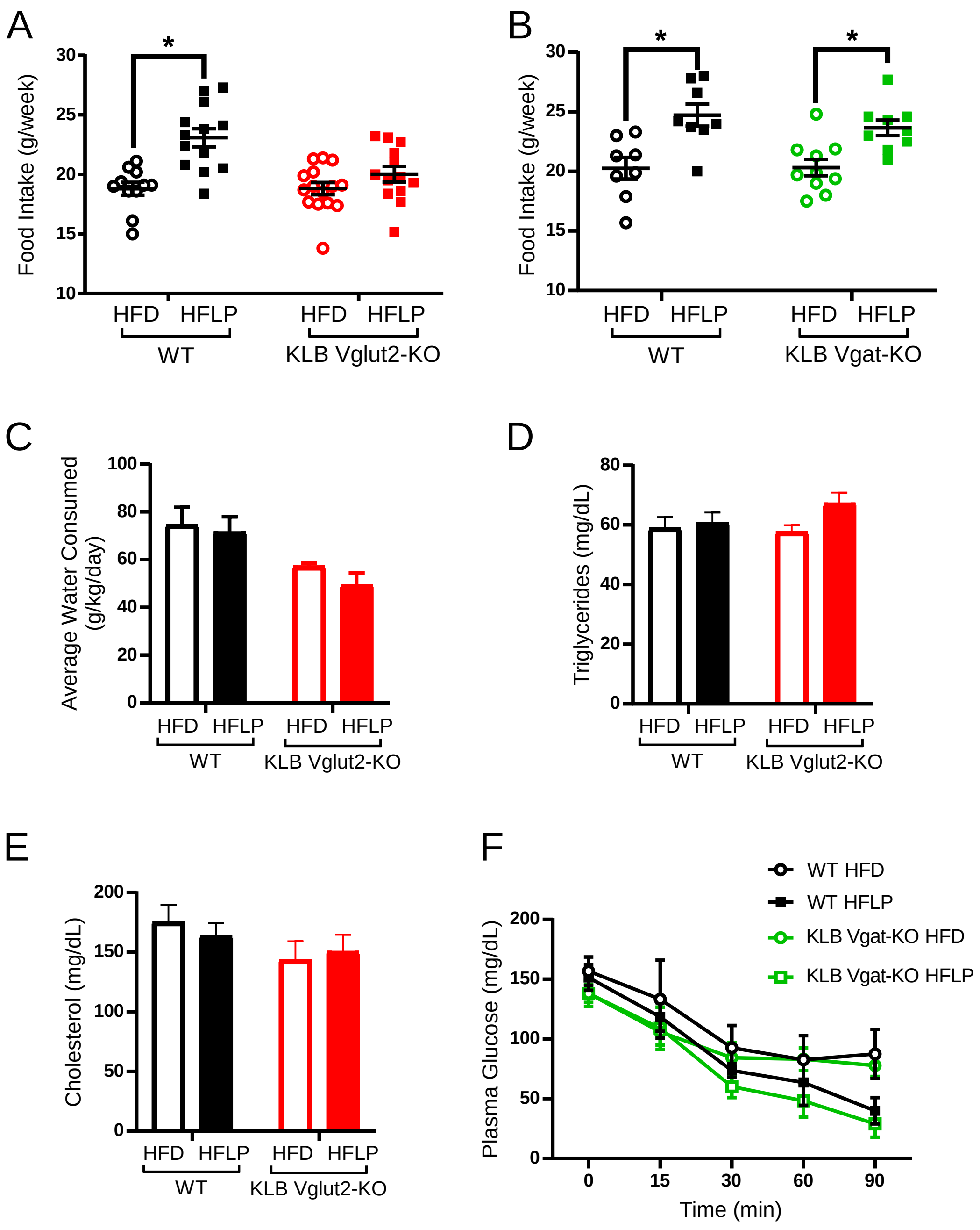

Supplementary Figure 2

Supplemental Table 1. Composition of diets related to methods.

D12450B

Class description	Ingredients	Grams	Caloric Information	
Protein	Casein, Lactic, 30 Mesh	200.00 g	Protein:	20 % Kcal
Protein	Cystine, L	3.00 g	Fat:	10 % Kcal
Carbohydrate	Sucrose, Fine Granulated	354.00 g	Carbohydrate:	70 % Kcal
Carbohydrate	Starch, Corn	315.00 g	Energy density:	3.82 Kcal/g
Carbohydrate	Lodex 10	35.00 g		
Fiber	Solka Floc, FCC200	50.00 g		
Fat	Soybean Oil, USP	25.00 g		
Fat	Lard	20.00 g		
Mineral	S10026B	50.00 g		
Vitamin	Choline Bitartrate	2.00 g		
Vitamin	V10001C	1.00 g		
Dye	Dye, Yellow FD&C #5, Alum. Lake 35-42%	0.05 g		

D12492

Class description	Ingredients	Grams	Caloric Information	
Protein	Casein, Lactic, 30 Mesh	200.00 g	Protein:	20 % Kcal
Protein	Cystine, L	3.00 g	Fat:	60 % Kcal
Carbohydrate	Lodex 10	125.00 g	Carbohydrate:	20 % Kcal
Carbohydrate	Sucrose, Fine Granulated	72.80 g	Energy density:	5.21 Kcal/g
Fiber	Solka Floc, FCC200	50.00 g		
Fat	Lard	245.00 g		
Fat	Soybean Oil, USP	25.00 g		
Mineral	S10026B	50.00 g		
Vitamin	Choline Bitartrate	2.00 g		
Vitamin	V10001C	1.00 g		
Dye	Dye, Blue FD&C #1, Alum. Lake 35-42%	0.05 g		

D10062201

Class description	Ingredients	Grams	Caloric Information	
Protein	Casein, Lactic, 30 Mesh	50.00 g	Protein:	5 % Kcal
Protein	Cystine, L	3.00 g	Fat:	10 % Kcal
Carbohydrate	Sucrose, Fine Granulated	354.00 g	Carbohydrate:	85 % Kcal
Carbohydrate	Starch, Corn	315.00 g	Energy density:	3.82 Kcal/g
Carbohydrate	Lodex 10	185.00 g		
Fiber	Solka Floc, FCC200	50.00 g		
Fat	Soybean Oil, USP	25.00 g		
Fat	Lard	20.00 g		
Mineral	S10026B	50.00 g		
Vitamin	Choline Bitartrate	2.00 g		
Vitamin	V10001C	1.00 g		
Dye	Dye, Red FD&C #40, Alum. Lake 35-42%	0.05 g		

D12020703

Class description	Ingredients	Grams	Caloric Information	
Protein	Casein, Lactic, 30 Mesh	50.00 g	Protein:	5 % Kcal
Protein	Cystine, L	3.00 g	Fat:	60 % Kcal
Carbohydrate	Lodex 10	325.00 g	Carbohydrate:	35 % Kcal
Carbohydrate	Sucrose, Fine Granulated	72.80 g	Energy density:	5.21 Kcal/g
Fiber	Solka Floc, FCC200	50.00 g		
Fat	Lard	245.00 g		
Fat	Soybean Oil, USP	25.00 g		
Mineral	S10026B	50.00 g		
Vitamin	Choline Bitartrate	2.00 g		
Vitamin	V10001C	1.00 g		
Dye	Dye, Red FD&C #40, Alum. Lake 35-42%	0.05 g		

Teklad 2920x

	Caloric Information
Protein:	19.1 % Kcal
Fat:	6.5 % Kcal
Carbohydrate:	47 % Kcal
Crude Fiber:	2.7 % Kcal
Neutral Detergent Fiber:	12.3% Kcal
Ash:	5.1 % Kcal
Energy density:	3.10 Kcal/g
